# Can Ultrasound Texture Analysis Differentiate Liver Metastases According to the Histopathological Origin of the Primary Tumor?

**DOI:** 10.3390/jcm15176815

**Published:** 2026-09-02

**Authors:** Seda Nida Karakucuk, Murat Baykara, Mehmet Demir, Ali İsler

**Affiliations:** 1 Department of Radiology, University Hospital, Kahramanmaras Sutcu Imam University, Kahramanmaraş 46040, Turkey; 2 Department of Radiology, Haydarpasa Numune Training and Research Hospital, University of Health Sciences, Istanbul 34384, Turkey; 3 Department of General Surgery, University Hospital, Kahramanmaras Sutcu Imam University, Kahramanmaraş 46040, Turkey

**Keywords:** liver metastasis, ultrasonography, texture analysis, tissue characterization, radiomics, histogram analysis

## Abstract

**Objective**: We aimed to investigate whether ultrasound-based texture analysis can differentiate liver metastases according to the histopathological origin of the primary tumor and to evaluate the quantitative texture characteristics of metastases originating from colorectal, pancreatic, and breast cancer. **Materials and Methods**: This prospective study included 75 patients with biopsy-proven liver metastases, comprising 25 colorectal adenocarcinoma, 25 pancreatic ductal adenocarcinoma, and 25 invasive ductal breast carcinoma metastases. Conventional B-mode ultrasound images were obtained prior to treatment. The largest metastatic lesion in each patient was manually segmented using a whole-lesion two-dimensional region of interest (ROI). Histogram-based texture analysis was performed using an in-house MATLAB-based software package (version R2021a; MathWorks, Natick, MA, USA). Extracted parameters included intensity-based metrics, dispersion measures, entropy, uniformity, and percentile values. Texture features were compared among the three groups using appropriate statistical tests. **Results**: Significant differences were observed among metastatic lesions according to their primary tumor origin. Significant differences were observed in the mean, median, minimum, maximum, most frequent gray-level values, root-mean-square level, root-sum-of-squares level, entropy, and all evaluated percentile parameters among groups (all *p* < 0.05). Pancreatic cancer metastases consistently demonstrated the highest intensity-related histogram values and percentiles, whereas breast cancer metastases exhibited the lowest values. Colorectal metastases were generally of intermediate intensity. Entropy values were significantly higher in colorectal and pancreatic metastases than in breast cancer metastases (*p* < 0.05), suggesting greater structural heterogeneity. No significant differences were observed for kurtosis, skewness, uniformity, or size distribution parameters (all *p* > 0.05). **Conclusions**: Ultrasound-based tissue analysis revealed distinct quantitative features among liver metastases originating from colorectal, pancreatic, and breast cancer. Density-related parameters, percentiles, and entropy show the potential to differentiate metastatic lesions based on their primary tumor origin, thus serving as a non-invasive biomarker.

## 1. Introduction

The liver is one of the most common sites of metastatic dissemination and is frequently involved in a wide range of primary malignancies, including colorectal, breast, pancreatic, lung, and gastric cancer [1,2,3,4,5,6]. While determining the primary source of liver metastases is often possible through clinical history, serum tumor markers, and contrast-enhanced CT or MRI, clinical practice may not always allow for it. Patients presenting with liver metastases of unknown primary origin, those with a history of multiple primary malignancies, and those with contraindications to contrast media or MRI (e.g., renal failure or severe contrast allergy) are more difficult to diagnose. Percutaneous liver biopsy is considered the gold standard method for confirming the primary source of atypical lesions [7]; however, tissue biopsies are limited by their inherently invasive nature, the risks of complications such as bleeding or tumor spread, and sampling errors due to intratumoral heterogeneity [8,9]. Therefore, there is growing interest in non-invasive, reproducible, and quantitative imaging biomarkers, such as texture analysis, that may provide additional information for characterizing liver metastases according to their primary tumor origin.

Tumor heterogeneity is increasingly recognized as a hallmark of cancer biology and has been associated with variations in cellularity, vascularity, necrosis, stromal composition, and treatment response [10]. These microscopic differences may influence imaging results and could potentially be quantified using texture analysis techniques. Tissue texture analysis is a promising quantitative imaging approach that objectively characterizes tissue heterogeneity through pixel-based features, providing information beyond conventional visual interpretation [11,12]. Ganeshan et al. reported that CT texture features could detect subtle tissue alterations associated with hepatic malignancy, even in apparently normal liver parenchyma [13]. Similarly, MRI-based texture analysis has shown potential for characterizing hepatic tumors and predicting histopathological features associated with tumor grade, biological behavior, and aggressiveness [14]. Because metastatic lesions may retain the histopathological and microenvironmental characteristics of their primary tumors, differences in stromal composition, glandular architecture, cellular density, and tumor growth patterns may contribute to differences in their imaging appearance. Colorectal adenocarcinoma, pancreatic ductal adenocarcinoma, and invasive ductal carcinoma of the breast represent distinct histopathological entities with well-recognized differences in tumor microenvironment and stromal organization [15]. These characteristics may contribute to measurable differences in ultrasound texture patterns.

Ultrasonography is routinely used as a first-line imaging modality to evaluate focal liver lesions because of its wide availability, low cost, and real-time imaging capability, making it readily applicable in daily clinical practice [16]. However, sonographic evaluation remains largely subjective, and the potential contribution of quantitative texture analysis to the characterization of hepatic metastases has not yet been fully explored.

Therefore, this study aimed to investigate the texture characteristics of liver metastases originating from colorectal, breast, and pancreatic cancer on conventional ultrasound images and to evaluate whether quantitative texture features differ according to their primary tumor origin.

## 2. Materials and Methods

### 2.1. Study Population

This prospective study was approved by the Institutional Ethics Committee (approval number/date 05/2023) and conducted in accordance with the principles of the Declaration of Helsinki. The study was conducted between October 2023 and September 2025. Written informed consent was obtained from all participants prior to enrollment.

Patients were prospectively recruited through referral from the General Surgery outpatient clinic with histopathologically confirmed colorectal adenocarcinoma, pancreatic ductal adenocarcinoma, or invasive ductal carcinoma of the breast, as well as biopsy-proven liver metastases. Only patients aged 18 years or older were included in the study.

Liver metastasis was confirmed by ultrasound-guided percutaneous liver biopsy in all patients. Histopathological evaluation served as the reference standard for diagnosis and determining primary tumor origin.

Patients with moderate-to-severe hepatic steatosis (grade 2–3), advanced ascites, previous local treatment of liver metastases, lesions smaller than 10 mm (considered insufficient in size for reliable ROI delineation and quantitative texture analysis), poor image quality, or deeply located lesions with indistinct margins that precluded accurate ROI delineation were excluded from the study. In patients with multiple metastatic lesions, the largest lesion was selected for analysis. None of the patients had received systemic chemotherapy before the ultrasound examination; this was initiated following the initial diagnosis.

**Figure 1:**
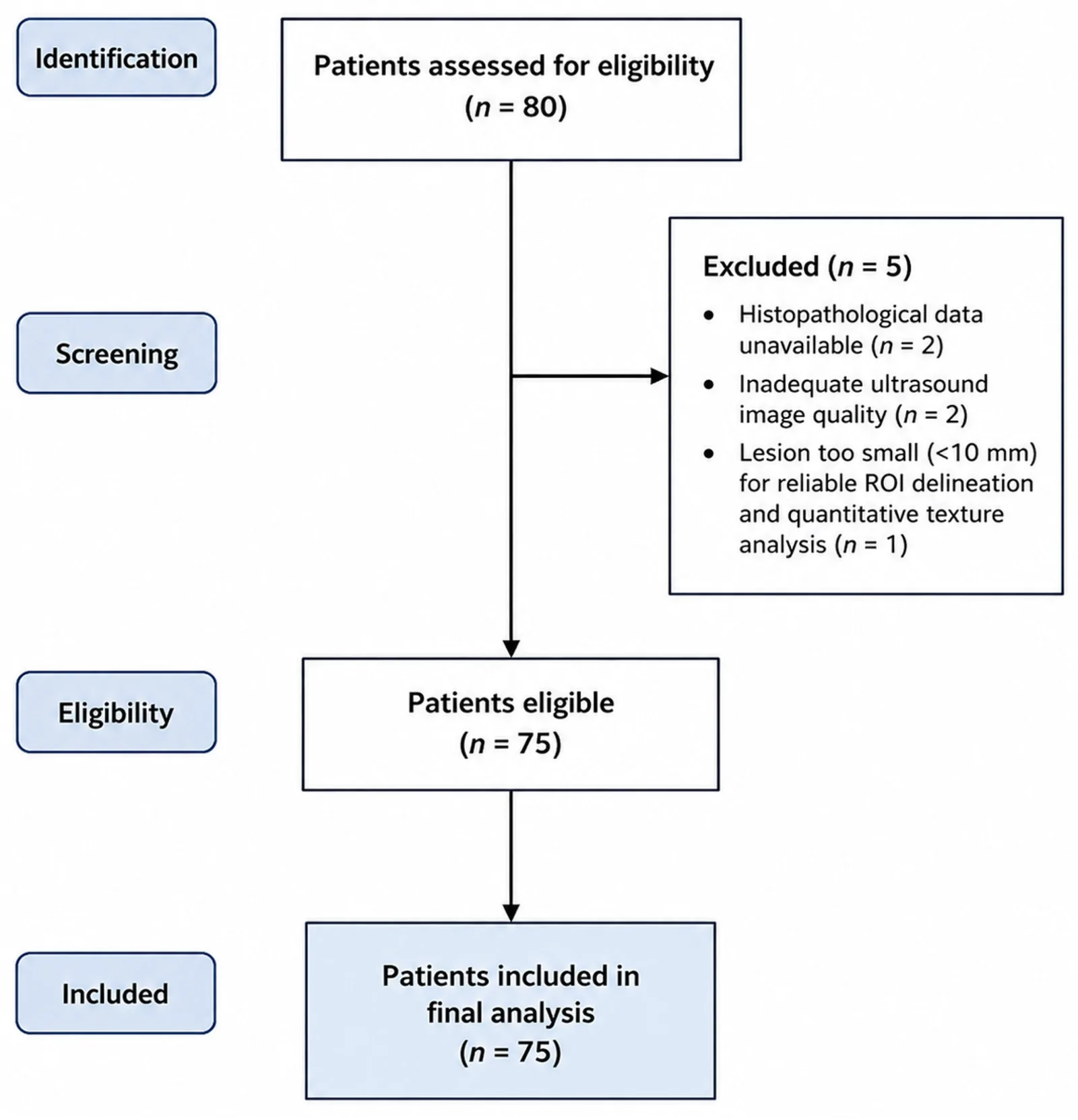
Study flow diagram of patient selection.

During the study period, 80 patients were assessed for eligibility. Five patients were excluded: two because histopathological data were unavailable, two because of inadequate ultrasound image quality, and one because the lesion was smaller than 10 mm and considered insufficient for reliable ROI delineation and quantitative texture analysis. A total of 75 patients were ultimately included in the final analysis. The patient selection process is illustrated in the study flow diagram (Figure 1).

### 2.2. Ultrasonographic Examination

All ultrasonographic examinations were performed using a LOGIQ E9 ultrasound system (GE Healthcare, Milwaukee, WI, USA) equipped with a C1–6 convex transducer. Examinations were performed by a radiologist with 16 years of experience in abdominal ultrasonography after at least 6 h of fasting. A routine abdominal ultrasound acquisition protocol was used, with a frequency of 4.0 MHz, an imaging depth of 12.0 cm, a dynamic range of 66 dB, and an acoustic output of 100%.

Conventional B-mode images of metastatic liver lesions were obtained and stored in Digital Imaging and Communications in Medicine (DICOM) format for subsequent analysis. For each patient, the image demonstrating the largest cross-sectional area of the metastatic lesion was selected.

### 2.3. Texture Analysis

Texture analysis was performed by a single radiologist with experience in abdominal ultrasonography who was blinded to the primary tumor type and clinical information. Conventional B-mode ultrasound images were transferred to a dedicated workstation for post-processing.

**Figure 2:**
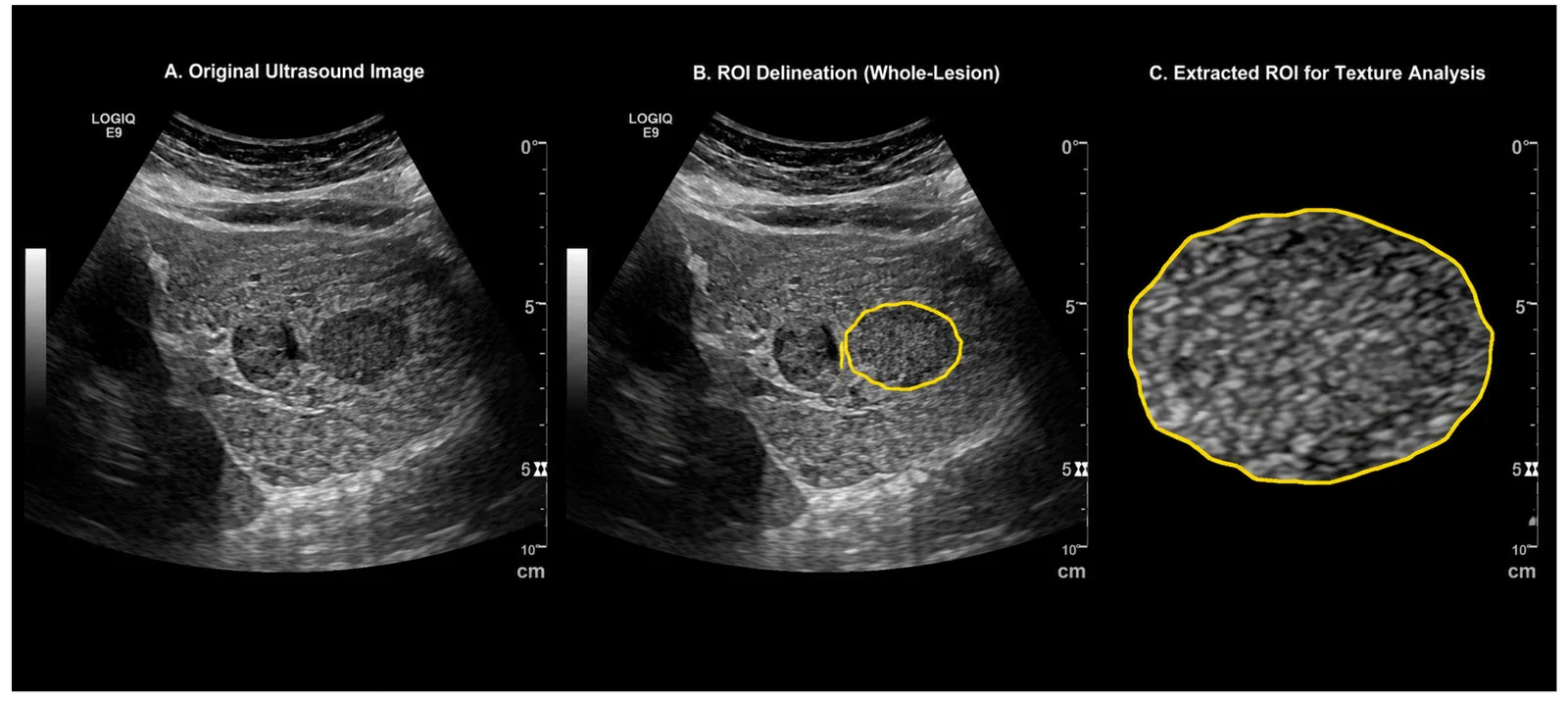
Ultrasound image acquisition and ROI-based texture analysis of a hepatic metastatic lesion. Representative grayscale ultrasound image of a liver metastasis (left), manual delineation of the lesion by whole-lesion ROI segmentation (middle), and isolated ROI used for texture feature extraction and quantitative tissue analysis (right).

For each patient, the largest metastatic liver lesion was selected for analysis. A two-dimensional (2D) region of interest (ROI) was manually delineated along the outer margin of the entire lesion on the image that demonstrated the largest cross-sectional tumor area. Care was taken to include the whole lesion while excluding adjacent liver parenchyma, large vessels, and image artifacts (Figure 2).

ROI segmentation was performed using a dedicated workstation (27-inch iMac, Apple Inc., Cupertino, CA, USA). Pixel data obtained from the segmented lesions were exported as XML (eXtensible Markup Language) files. Texture analysis was subsequently performed on Windows 10 (Microsoft Corporation, Redmond, WA, USA) using an in-house MATLAB-based software package (version R2021a; MathWorks, Natick, MA, USA).

Histogram-based texture features were extracted from each ROI. The evaluated parameters included mean, standard deviation, median, mean absolute deviation, median absolute deviation, minimum, maximum, variance, range, interquartile range, most frequent value, Size %L, Size %M, Size %U, kurtosis, skewness, smoothness, root-mean-square level, root-sum-of-squares level, 1st, 3rd, 5th, 10th, 25th, 75th, 90th, 95th, 97th, and 99th percentiles, as well as entropy and uniformity. The analysis was based exclusively on first-order histogram-based features describing the statistical distribution of grayscale pixel intensities within the ROI. These parameters were calculated directly from the grayscale pixel-intensity distribution using standard statistical definitions. No second-order or higher-order spatial texture matrices, including the gray-level co-occurrence matrix (GLCM) or the gray-level run-length matrix (GLRLM), were used. Accordingly, the analysis did not incorporate spatial relationships between neighboring pixels.

These texture parameters were used to quantitatively assess lesion heterogeneity and to compare liver metastases originating from breast, colorectal, and pancreatic cancer.

### 2.4. Statistical Analysis

Continuous variables were expressed as mean ± standard deviation or median (minimum–maximum), as appropriate, and categorical variables were presented as frequencies and percentages. Normality was assessed using the Kolmogorov–Smirnov and Shapiro–Wilk tests. Comparisons among the three groups were performed using one-way ANOVA for normally distributed variables and the Kruskal–Wallis test for non-normally distributed variables. Post hoc pairwise comparisons were performed using the Mann–Whitney U test. Categorical variables were compared using the Chi-square test. Statistical analyses were performed using SPSS version 28.0 (IBM Corp. Armonk, NY, USA). A *p* value < 0.05 was considered statistically significant.

### 2.5. Use of Generative Artificial İntelligence

During the preparation of this manuscript, ChatGPT (OpenAl, GPT-5.5) grammar checking and language editing to improve the English language and readability of the manuscript. The AI tool was not used to generate scientific content, analyze or interpret data, or draw scientific conclusions. The authors reviewed and edited the content as necessary and take full responsibility for the final content of the manuscript.

## 3. Results

A total of 75 patients were included in the study, comprising 25 patients each with liver metastases from colorectal adenocarcinoma, pancreatic ductal adenocarcinoma, and invasive ductal breast carcinoma. The baseline characteristics of the metastases were also evaluated. Among the 75 patients included in the study, lesions were located predominantly in the right hepatic lobe (58 patients, 77.3%), while 17 patients (22.7%) had lesions in the left hepatic lobe. Solitary lesions were present in 17 patients (22.7%), whereas 58 (77.3%) had multiple lesions. The mean lesion size was 25.4 ± 8.3 mm, and the mean depth was 35.6 ± 11.1 mm.

There was no significant difference in age among the three groups (*p* = 0.073). The ages of the patients included in the study ranged from 49.0 to 92.0 years, with a median of 69.0 years and an average of 66.9 ± 9.1 years. As expected, sex distribution differed significantly between groups (*p* < 0.001), with all patients in the breast cancer metastasis group being female (Table 1).

Histogram-based texture analysis demonstrated significant differences among metastatic lesions according to their primary tumor origin (Table 1). The mean, median, minimum, maximum, and most frequent histogram values differed significantly between groups (all *p* < 0.001). Post hoc analyses revealed that pancreatic cancer metastases exhibited significantly higher values for these parameters compared with both colorectal and breast cancer metastases. In addition, colorectal cancer metastases showed significantly higher values than breast cancer metastases in their mean, median, maximum, and most frequent histogram.

Among histogram dispersion metrics, only the range demonstrated a significant difference among groups (*p* = 0.015), with colorectal and pancreatic metastases showing higher values than breast cancer metastases. Standard deviation, mean absolute deviation, median absolute deviation, variance, and interquartile range did not differ significantly among groups (all *p* > 0.05).

No significant differences were observed for histogram kurtosis, skewness, smoothness, uniformity, or size distribution parameters (%L, %M, and %U) (all *p* > 0.05) (Table 2).

Root-mean-square level and root-sum-of-squares level differed significantly among groups (both *p* < 0.001). Pancreatic metastases demonstrated the highest values, followed by colorectal and breast cancer metastases, which exhibited the lowest values.

Percentile-based histogram parameters, including the 1st, 3rd, 5th, 10th, 25th, 75th, 90th, 95th, 97th, and 99th percentiles, showed significant differences among groups (all *p* < 0.001) (Table 2). Pancreatic metastases consistently demonstrated higher percentile values than both colorectal and breast cancer metastases, while those of colorectal metastases were generally higher than breast cancer metastases.

Entropy also differed significantly among groups (*p* = 0.014). Both colorectal and pancreatic metastases demonstrated higher entropy values than breast cancer metastases, whereas no significant difference was observed between colorectal and pancreatic metastases.

Overall, liver metastases originating from pancreatic cancer exhibited the highest gray-level intensity-related texture parameters, breast cancer metastases exhibited the lowest values, and colorectal cancer metastases generally exhibited intermediate characteristics.

## 4. Discussion

The principal finding of this study is that quantitative ultrasound tissue analysis revealed significant differences in the imaging characteristics of liver metastases according to the histopathological origin of the primary tumor. These findings suggest that routinely acquired B-mode ultrasound images contain quantitative information that may reflect differences in the underlying structural characteristics of metastatic lesions. Thus, ultrasound-based tissue analysis may provide complementary information to conventional sonographic assessment and have potential as a non-invasive approach for the characterization of liver metastases.

The concept that quantitative imaging features reflect underlying tumor biology has been increasingly supported by research in radiomics. Previous CT- and MRI-based studies have demonstrated that texture features are associated with tumor grade, vascularity, treatment response, and survival outcomes [17,18,19]. In particular, texture analysis of colorectal liver metastases has shown that imaging-derived heterogeneity metrics correlate with pathological findings and clinical prognosis. These findings suggest that quantitative texture parameters may reflect underlying differences in tumor microstructure and biological characteristics [20].

In a previous study, Becker et al. demonstrated that histogram and grayscale parameters could be used to detect liver metastases in mice [21]. They then compared patients with and without colorectal metastases in the liver, showing that histogram parameters could be used to predict the presence of metastases at an early stage [22].

The significantly elevated grayscale density and percentage values observed in pancreatic ductal adenocarcinoma metastases may be related to the specific tumor microenvironment of this malignancy, which is characterized by a desmoplastic stromal reaction and extracellular matrix composition. These histopathological features can affect acoustic backscatter properties and lead to different ultrasound appearances of metastatic lesions [23,24]. More broadly, interactions between tumors and their surrounding biological environment may contribute to differences in disease phenotypes [25]. In pancreatic cancer, the tumor microenvironment is influenced by complex interactions involving intestinal homeostasis, the microbiota, and immune mechanisms, which may contribute to tumor behavior [26]. The higher grayscale values observed in pancreatic ductal adenocarcinoma metastases may reflect differences in the biological and microenvironmental characteristics of these lesions.

In contrast, our study showed that liver metastases from breast cancer exhibited the lowest gray-level intensity parameters and percentiles. From a histopathological perspective, differences in cellularity, stromal composition, and necrotic components may contribute to variations in the acoustic characteristics of metastatic lesions. Our results also revealed that colorectal cancer metastases occupied an intermediate position in terms of density characteristics, but showed significantly higher entropy and mean values compared to breast cancer metastases. Entropy is a statistical measure of image complexity or randomness and may reflect structural tumor heterogeneity within a lesion [27]. Higher entropy values have been associated with increased structural disorder and biological aggressiveness in various malignancies [23,28].

Importantly, the present study specifically evaluated liver metastases originating from colorectal adenocarcinoma, pancreatic ductal adenocarcinoma, and invasive ductal carcinoma of the breast, which represent the most common histological subtypes of these malignancies. Therefore, the identified texture signatures should be interpreted within the context of these specific histopathological subtypes rather than generalized to all colorectal, pancreatic, or breast malignancies. While many previous studies have successfully used CT- and MR-based radiographic methods to characterize focal liver and pancreatic lesions and predict treatment responses [29], the application of quantitative tissue analysis to conventional B-mode ultrasonography is relatively limited. Ultrasonography is a commonly preferred imaging modality for the evaluation of focal liver lesions in clinical practice. Our study suggests that, by converting standard B-mode images into reproducible, objective pixel-level datasets, ultrasound tissue analysis may provide additional quantitative information that could help differentiate liver metastases according to the histopathological origin of the primary tumor.

Clinically, the value of quantitative ultrasound tissue analysis may be quite significant, particularly in cases where the primary origin of liver metastasis remains unclear despite current clinical and imaging information, such as in patients with multiple possible primary malignancies or where contrast-enhanced CT or MRI is not readily available. While histopathological confirmation is essential, quantitative analysis of routinely acquired B-mode ultrasound images can provide additional objective information for lesion characterization and potentially complement conventional diagnostic investigation in these situations.

Several limitations of this study should be acknowledged. First, this was a single-center study with a relatively small sample size, which may restrict the generalizability of the findings. Larger, multicenter studies are needed to confirm the reproducibility and robustness of the observed texture differences.

Second, texture analysis was based on a single two-dimensional (2D) region of interest (ROI) obtained from the largest cross-sectional area of each lesion, rather than on whole-lesion three-dimensional (3D) analysis. In patients with multiple metastases, only the largest eligible lesion was analyzed. Although this one-patient/one-lesion approach facilitated standardized ROI delineation and avoided statistical dependence between multiple lesions, it may not fully reflect lesion-to-lesion or intralesional heterogeneity. Future studies should evaluate multiple lesions and/or whole-lesion 3D volumetric analysis using appropriate methods to account for within-patient correlation.

Third, all ultrasound examinations and ROI delineations were performed by a single experienced radiologist; therefore, interobserver variability and reproducibility were not assessed. Studies involving multiple observers are needed to evaluate the robustness of the methodology.

Fourth, the study was designed to compare quantitative ultrasound features among predefined histopathological groups rather than to develop a predictive classification model. Thus, cross-validation and external validation were not performed, and the findings should be considered exploratory and hypothesis-generating. Future studies should validate these findings in independent cohorts and incorporate appropriate internal and external validation.

Fifth, the analysis was limited to first-order histogram-based features, which describe intensity distributions but do not capture spatial relationships between neighboring pixels. Higher-order texture or radiomics features, as well as machine-learning or deep-learning approaches, may provide additional information and improve the discrimination of metastatic lesions according to primary tumor origin.

Sixth, the exclusion of patients with moderate-to-severe hepatic steatosis, advanced ascites, previous local treatment, deeply located or small lesions, and inadequate image quality resulted in a relatively selected population. Although necessary for reliable image analysis, these criteria may introduce selection bias and limit generalizability to the broader population of patients with liver metastases.

Finally, only colorectal adenocarcinoma, pancreatic ductal adenocarcinoma, and invasive ductal carcinoma of the breast were included. Other histopathological variants were not represented; therefore, the findings may not be generalizable to all liver metastases originating from these primary malignancies.

In conclusion, ultrasound-based tissue analysis demonstrated significant differences among liver metastases originating from colorectal, pancreatic, and breast cancers. Among histogram parameters, gray level density, percentiles, and entropy showed potential for distinguishing metastatic lesions according to their primary tumor origins. These findings suggest that quantitative ultrasound tissue analysis may provide a useful, non-invasive imaging biomarker for the characterization of liver metastases and may warrant further validation in larger, multicenter studies.

## Figures and Tables

**Table 1:** Demographic characteristics and first-order histogram texture features of liver metastases according to primary tumor origin.

			Organ Metastasis									*p*	
			Colon (*n*: 25) ^1^			Pancreas (*n*: 25) ^2^			Breast (*n:* 25) ^3^				
Age	Mean ± sd		67.6	±	12.3	69.3	±	5.3	63.7	±	7.7	0.073	^K^
	Median		70.0			69.0			61.0				
Gender	Male	*n*-%	19		76.0%	16		64.0%	0		0.0%	0.000	^X2^
	Female	*n*-%	6 ^3^		24.0%	9 ^3^		36.0%	25		100%		
Mean of Histogram	Mean ± sd		76.7	±	13.7	123.5	±	18.4	55.5	±	20.5	0.000	^K^
	Median		80.7 ^2^			123.7			56.6 ^1,2^				
Standard Deviation of Histogram	Mean ± sd		19.8	±	5.0	19.2	±	5.0	17.1	±	5.2	0.136	^K^
	Median		18.4			20.3			15.6				
Median of Histogram	Mean ± sd		76.0	±	13.8	123.1	±	18.6	55.4	±	21.0	0.000	^K^
	Median		80.0 ^2^			121.0			58.0 ^1,2^				
Minimum of Histogram	Mean ± sd		20.2	±	17.8	65.6	±	25.8	11.2	±	12.7	0.000	^K^
	Median		17.0 ^2^			66.0			5.0 ^2^				
Maximum of Histogram	Mean ± sd		156.8	±	30.3	194.0	±	26.0	117.8	±	38.7	0.000	^K^
	Median		154.0 ^2^			201.0			120.0 ^1,2^				
Mean Absolute Deviation of Histogram	Mean ± sd		15.7	±	4.1	15.5	±	4.0	13.8	±	4.2	0.167	^K^
	Median		14.9			16.2			12.8				
Median Absolute Deviation of Histogram	Mean ± sd		13.1	±	3.9	13.3	±	3.4	11.8	±	3.6	0.197	^K^
	Median		13.0			14.0			11.0				
Variance of Histogram	Mean ± sd		415.1	±	235.0	393.8	±	190.1	319.1	±	194.8	0.136	^K^
	Median		340.1			410.3			243.8				
Range of Histogram	Mean ± sd		136.6	±	36.2	128.3	±	36.7	106.6	±	37.1	0.015	^K^
	Median		124.0			132.0			89.0 ^1,2^				
Interquartile Range of Histogram	Mean ± sd		26.1	±	7.8	26.7	±	6.8	23.5	±	7.4	0.199	^K^
	Median		25.0			27.0			21.0				
Most Frequent Value of Histogram	Mean ± sd		71.9	±	14.4	125.7	±	19.9	53.4	±	22.4	0.000	^K^
	Median		79.0 ^2^			126.0			58.0 ^1,2^				
Size %L of Histogram	Mean ± sd		15.7	±	2.2	16.6	±	1.3	16.4	±	1.7	0.255	^K^
	Median		16.0			16.7			16.6				
Size %M of Histogram	Mean ± sd		68.9	±	3.2	67.3	±	2.1	68.3	±	2.2	0.088	^A^
	Median		68.7			67.1			68.4				
Size %U of Histogram	Mean ± sd		15.4	±	1.4	16.1	±	1.3	15.4	±	1.3	0.096	^A^
	Median		15.5			16.3			15.4				

^A^ ANOVA/^K^ Kruskal–Wallis (Mann–Whitney u test)/^X2^ Chi-square test; Difference with ^1^ Colon Group *p* < 0.05; Difference with ^2^ Pancreas Group *p* < 0.05; ^3^ Breast Group *p* < 0.05.

**Table 2:** Higher-order histogram descriptors, percentile-based parameters, and heterogeneity metrics of liver metastases according to primary tumor origin.

			Organ Metastasis									*p*	
			Colon (*n*: 25) ^1^			Pancreas (*n:* 25) ^2^			Breast (*n*: 25) ^3^				
Kurtosis of Histogram	Mean ± sd		3.37	±	0.85	2.96	±	0.57	2.99	±	0.54	0.063	^K^
	Median		3.04			2.85			2.91				
Skewness of Histogram	Mean ± sd		0.27	±	0.36	0.10	±	0.32	0.20	±	0.28	0.167	^A^
	Median		0.19			0.10			0.13				
Smoothness of Histogram	Mean ± sd		0.003	±	0.001	0.003	±	0.002	0.004	±	0.002	0.136	^K^
	Median		0.003			0.002			0.004				
Root-Mean-Square Level of Histogram	Mean ± sd		79.4	±	13.4	125.1	±	18.3	58.3	±	20.5	0.000	^K^
	Median		82.1 ^2^			125.8			58.2 ^1,2^				
Root-Sum-of-Squares Level of Histogram	Mean ± sd		103.01	±	47.45	180.53	±	117.37	51.17	±	32.23	0.000	^K^
	Median		101.51 ^2^			143.25			53.92 ^1,2^				
1st Percentile of Histogram	Mean ± sd		34.6	±	17.1	81.3	±	20.8	19.9	±	16.2	0.000	^K^
	Median		36.0 ^2^			78.0			17.0 ^1,2^				
3rd Percentile of Histogram	Mean ± sd		41.4	±	16.4	87.9	±	19.9	25.1	±	16.3	0.000	^K^
	Median		42.0 ^2^			86.0			21.0 ^1,2^				
5th Percentile of Histogram	Mean ± sd		45.4	±	15.6	91.8	±	19.4	28.0	±	17.1	0.000	^K^
	Median		46.0 ^2^			90.0			24.0 ^1,2^				
10th Percentile of Histogram	Mean ± sd		52.0	±	14.9	98.3	±	18.4	33.5	±	17.1	0.000	^K^
	Median		50.0 ^2^			98.0			30.0 ^1,2^				
25th Percentile of Histogram	Mean ± sd		63.3	±	14.4	109.8	±	18.6	43.4	±	18.4	0.000	^K^
	Median		63.0 ^2^			110.0			48.0 ^1,2^				
75th Percentile of Histogram	Mean ± sd		89.4	±	14.6	136.4	±	19.4	66.9	±	22.8	0.000	^A^
	Median		90.0 ^2^			135.0			66.0 ^1,2^				
90th Percentile of Histogram	Mean ± sd		102.0	±	15.7	148.2	±	20.1	77.9	±	24.9	0.000	^A^
	Median		103.0 ^2^			148.0			77.0 ^1,2^				
95th Percentile of Histogram	Mean ± sd		110.4	±	16.6	154.8	±	20.4	84.4	±	26.6	0.000	^A^
	Median		110.0 ^2^			153.0			80.0 ^1,2^				
97th Percentile of Histogram	Mean ± sd		115.9	±	17.7	159.1	±	21.2	88.8	±	27.8	0.000	^A^
	Median		117.0 ^2^			156.0			84.0 ^1,2^				
99th Percentile of Histogram	Mean ± sd		126.9	±	19.4	167.3	±	21.8	97.0	±	30.9	0.000	^A^
	Median		129.0 ^2^			162.0			92.0 ^1,2^				
Entropy of Histogram	Mean ± sd		5.69	±	0.32	5.77	±	0.41	5.40	±	0.52	0.014	^K^
	Median		5.68			5.82			5.36 ^1,2^				
Uniformity of Histogram	Mean ± sd		0.20	±	0.06	0.23	±	0.06	0.21	±	0.07	0.337	^A^
	Median		0.20			0.23			0.21				

^A^ ANOVA/^K^ Kruskal–Wallis (Mann–Whitney u test); Difference with ^1^ Colon Group *p* < 0.05; Difference with ^2^ Pancreas Group *p* < 0.05; ^3^ Breast Group *p* < 0.05.

## Data Availability

The data presented in this study are available on request from the corresponding author.
